# Multidimensional disparity in inadequate minimum dietary diversity between poor and non-poor children aged 6–23 months in Sub-Saharan Africa: a multivariate decomposition analysis

**DOI:** 10.3389/fpubh.2025.1516129

**Published:** 2025-04-02

**Authors:** Abel Endawkie, Yawkal Tsega, Desale B. Asmamaw, Natnael Kebede, Mastewal Arefaynie, Temeselew Woldetsadik Mawugatie

**Affiliations:** ^1^Department of Epidemiology and Biostatistics, School of Public Health, College of Medicine and Health Sciences, Wollo University, Dessie, Ethiopia; ^2^Department of Health System and Management School of Public Health, College of Medicine and Health Sciences, Wollo University, Dessie, Ethiopia; ^3^Department of Reproductive Health, College of Medicine and Health Sciences, Institute of Public Health, University of Gondar, Gondar, Ethiopia; ^4^Monash Centre for Health Research and Implementation, Faculty of Medicine, Nursing and Health Sciences, Monash University, Melbourne, VIC, Australia; ^5^Department of Health Promotion, School of Public Health College of Medicine and Health Sciences, Wollo University, Dessie, Ethiopia; ^6^Department of Reproductive and Family Health, School of Public Health, College of Medicine and Health Sciences, Wollo University, Dessie, Ethiopia; ^7^Department of Economics, College of Business and Economics, Wollo University, Dessie, Ethiopia

**Keywords:** multivariate decomposition, multidimensional poor-non-poor, minimum dietary diversity, children, Sub-Saharan Africa

## Abstract

**Background:**

Sustainable Development Goal (SDG) 2 aims to “end hunger, achieve food security, and improve nutrition” by 2030. However, the prevalence of inadequate Minimum Dietary Diversity (MDD) is on the rise in Sub-Saharan Africa (SSA). Therefore, this study aimed to assess the disparities between multidimensional poor and non-poor households in terms of inadequate MDD among children aged 6 to 23 months in SSA, using data from the 2018–2023 Demographic and Health Survey (DHS).

**Methods:**

The study utilized data from a nationally representative weighted sample of 352,463 children aged 6 to 23 months, drawn from the latest rounds of the DHS in 18 SSA countries. A decomposition analysis was performed to assess the disparity in inadequate MDD between multidimensionally poor and non-poor Households. This analysis divided the disparity into two components: one related to differences in the levels of determinants (endowments) between the poor and non-poor, and the other concerning variations in the effects of the covariates.

**Results:**

The overall prevalence of inadequate MDD among children in SSA was 89.05%. This prevalence was highest in Central Africa at 90.55% and lowest in South Africa at 87.8%. The difference in inadequate MDD between multidimensional poor and non-poor children was highest in East Africa at 6.15%, which was statistically significant. Factors such as women’s education, husband’s educational status, the employment status of both parents, household wealth index, place of residence, family size, and the number of children significantly contributed to the disparity in inadequate MDD among children from multidimensional poor and non-poor households in SSA.

**Conclusion:**

The study highlights a concerningly high prevalence of inadequate MDD among children in SSA, with significant disparities observed between multidimensionally poor and non-poor households. The largest gap in inadequate MDD between poor and non-poor households was found in East Africa. Key factors contributing to these disparities include women’s education, husband’s educational status, the employment status of both parents, household wealth index, place of residence (urban vs. rural), family size, and the number of children. The findings underscore the urgent need for targeted interventions to address inadequate MDD among young children in SSA. Efforts should focus on reducing poverty, improving maternal education, and enhancing employment opportunities, particularly for women, by promoting equitable economic prospects. Addressing these underlying factors is essential to closing the gap in dietary diversity and improving the nutritional outcomes of children in the region.

## Introduction

Inadequate feeding practices are a primary cause of childhood malnutrition, and their repercussions are among the most serious roadblocks to long-term socioeconomic growth and poverty reduction worldwide (1, 2). Inadequate MDD is most common in low- and middle-income countries, where it has a significant impact on increasing mortality and disease distribution (3–5). The prevalence of inadequate MDD is high (6), especially in SSA.

Recently, poor nutrition has been responsible for 45% of under-five mortality, with South Asia and Sub-Saharan Africa bearing the highest burden (7). Between the ages of 6 and 23 months, children’s nutritional needs per body weight increase, rendering breast milk insufficient to meet all their needs (8). After 6 months, children should receive adequate and acceptable supplemental foods while continuing to lactate for at least 2 years (9).

Various explanatory variables, such as women’s education (1, 10, 11), household wealth index (1, 10, 11), antenatal care (12, 13), father’s educational status (1, 10, 11, 13), child age (1, 14), mass media exposure (1, 10, 11), maternal age (14, 15), occupational status (16, 17), and place of residence (17) in different countries, were found to be significantly associated with MDD among children aged 6–23 months in several studies.

Inadequate MDD is defined as a child who does not consume a minimum of five food groups out of a total of eight. The eight food groups include breast milk, grains, roots and tubers, legumes and nuts, dairy products, flesh foods (such as meat, fish, poultry, and liver or organ meats), eggs, vitamin A-rich fruits and vegetables, as well as other fruits and vegetables (18). The World Health Organization (WHO) (9) and the United Nations International Children’s Emergency Fund (UNICEF) (19) are currently focusing on children’s nutritional issues. The Sustainable Development Goals (SDGs) for 2015–2030 were also established, with objectives including eradicating extreme poverty and hunger while enhancing health and education (20). Specifically, SDG 2 aims to “end hunger, achieve food security and improve nutrition (21), and SDG 3 mainly focuses on “leaving no one behind in accessing essential health services “by 2030 (21–23).

However, in Sub-Saharan Africa, situations are contrary, with SSA facing significant challenges in achieving this goal (24). According to statistics, food insecurity in SSA has increased since 2015 (24, 25). Sub-Saharan Africa has pervasive disparities in living standards, health, and education throughout all of its states and geopolitical regions (23, 24). Traditionally, poverty has been measured using thresholds for one-dimensional indicators such as household wealth index or consumption, which do not capture what it means for people of different age groups to be poor, particularly children (14, 26). Therefore, this study incorporates the multidimensional measure of poverty to precisely represent the range of sufferings. Multidimensional poverty was assessed using criteria published by the UNDP’s Human Development Report Office (27), which considers three dimensions: health, education, and living standards (28). However, the decomposition analysis method is useful for examining inequalities in inadequate MDD between multidimensional non-poor and poor households. The disparity between these groups has not been thoroughly explored in the context of SSA. Thus, this study aims to determine the disparity in inadequate MDD among children aged 6–23 months in SSA using the recent 2018–2023 Demographic and Health Survey (DHS) data. This evidence is particularly important for informing the design of programs and policies at the local, regional, and national levels to address the compositional and behavioral determinants contributing to disparities in inadequate MDD among children in multidimensional poor-non-poor households in Sub-Saharan Africa.

## Materials and methods

### Study setting and period

The study was conducted in Sub-Saharan Africa, a region of the African continent characterized by its diverse population. In this study, the 18 selected countries of SSA were divided into four regions: Central Africa (Burundi, Gabon, Guinea, and Côte d’Ivoire), East Africa (Ethiopia, Kenya, Tanzania, and Rwanda), Southern Africa (Madagascar, Mali, Zambia, and Mauritius), and West Africa (Burkina Faso, Gambia, Nigeria, Liberia, Senegal, and Sierra Leone). Sub-Saharan Africa is home to over 40 countries and approximately 1 billion people, showcasing a rich and varied culture. The research utilized the latest publicly available, nationally representative DHS data from 2018 to 2023, covering 18 countries in Sub-Saharan Africa.

### Study design

The research used a cross-sectional study design based on secondary data from the recent DHS in Sub-Saharan Africa.

### Source and study population

The source population included all children aged 6–23 months, and the study population was those in the selected Enumeration Areas (EAs) in Sub-Saharan Africa.

### Data source

We obtained the dependent and independent variables from the latest Demographic and Health Surveys (DHS) birth record (BR) dataset, which includes the complete birth history of all interviewed women. This dataset also provides health and nutrition information for children under 5 years old. The data used in this study represent the most recent nationally representative DHS data, specifically from 2018 to 2023 (see details in Table 1). During the merging process, duplicates were generated, and we established duplicate control by creating a country code and merging it with the first cluster number. We then removed a dot and ensured that the last digit of the country code differed from zero, allowing us to uniquely identify each cluster or enumeration area.

**Table 1:** The sample size of children aged 6–23 months included in this study from 18 SSA countries using Demographic and Health Surveys from 2018 to 2023.

Country code	Survey data set	Year	Country name	Sample size	Prevalence of inadequate MDD (%)
BG	Demographic and Health Survey	2018	Brundi	35,576	87
BF	Demographic and Health Survey	2021	Burkina Faso	15,147	99
CL	Demographic and Health Survey	2021	Cot divar	11,371	97
ET	Demographic and Health Survey	2019	Ethiopia	14,173	93
GA	Demographic and Health Survey	2021	Gabon	13,661	88
GM	Demographic and Health Survey	2020	Gambia	10,340	96
GN	Demographic and Health Survey	2018	Guinea	9,380	94
KE	Demographic and Health Survey	2022	Kenya	9,380	93
LB	Demographic and Health Survey	2020	Liberia	11,107	95
MD	Demographic and Health Survey	2021	Madagascar	29,068	91
ML	Demographic and Health Survey	2018	Mali	31,716	91
MR	Demographic and Health Survey	2021	Mauritius	16,143	95
NG	Demographic and Health Survey	2018	Nigeria	44,636	93
RW	Demographic and Health Survey	2020	Rwanda	28,897	83
SN	Demographic and Health Survey	2019	Senegal	14,616	89
SL	Demographic and Health Survey	2019	Sierra Leone	11,621	93
TZ	Demographic and Health Survey	2022	Tanzania	20,217	96
ZM	Demographic Health Survey	2018	Zambia	25,010	91

### Sample size and sampling method

The study includes weighted samples from 352,463 children aged 6–23 months, collected from approximately 1,692 enumeration areas across 62 regions or provinces in 18 Sub-Saharan African countries. The Demographic and Health Surveys (DHS) employed a two-stage stratified cluster sampling method. In the first stage, enumeration areas (EAs) were independently selected from each stratum with proportional allocation based on place of residence (urban and rural). In the second stage, households were systematically sampled from the chosen EAs.

### Variable measurement

#### Dependent variable

A child was considered to have inadequate MDD if they did not consume at least five of the eight food groups in the 24 h prior to the interview. The food groups include: (1) breast milk, (2) grains, roots, and tubers, (3) legumes and nuts, (4) dairy products, (5) flesh foods (such as meat, fish, poultry, and organ meats), (6) eggs, (7) vitamin A-rich fruits and vegetables, and (8) other fruits and vegetables (18). The MDD was categorized as “adequate” with a label of “0” and “inadequate” with a label of “1.”

Sociodemographic factors considered as independent variables include the age of the mother, father, or child; the sex of the household head; the education level of both parents; their employment status; household wealth index; place of residence; antenatal care; birth intervals; the number of living children; household size; and the region within Sub-Saharan Africa.

#### Equity stratifying variable

The Multidimensional Poverty Index (MPI) is used as an independent variable for stratifying and classifying individuals as non-poor (“0”) or poor (“1”). The MPI was measured based on the criteria established by the UNDP’s Human Development Report Office (28). The MPI measurement was based on 3 dimensions and 10 indicators.

Health: child mortality [yes, deprived (1) and non-deprived (0)], and nutrition {at least one household member who was sick and unable to perform normal activities in the last 4 weeks: yes [deprived (1) and non-deprived (0)]}.

Education: years of schooling [no household member aged 7–17 years or older has completed 5 years of schooling: deprived (1) and non-deprived (0)]; school attendance [any school-aged child not attending school up to age 8: deprived (1) and non-deprived (0)].

Living Standards: Water: Households using water from unimproved sources, such as open wells, springs, or surface water, are considered deprived (1), while those using improved sources are regarded as non-deprived (0). Sanitation/Toilet Facilities: Households utilizing unimproved sanitation facilities, such as pit latrines without slabs, open pit latrines, or hanging toilets, are classified as deprived (1), whereas those with improved facilities are considered non-deprived (0). Electricity: Households without electricity are deemed deprived (1), while those with electricity are recognized as non-deprived (0). Cooking Fuel: Households that cook with wood, charcoal, or dung are classified as deprived (1), while those using cleaner fuels are considered non-deprived (0). House occupancy status: Households that do not own their home are classified as deprived (1), while homeowners are considered non-deprived (0). Assets: Households lacking at least one of the following assets—radio, TV, mobile phone, tape recorder, or refrigerator—are considered deprived (1), while those owning at least one are regarded as non-deprived (0).

To determine the MPI, we adopt the recommended poverty threshold based on standard dimensions and their indicators (29). Each individual is assigned a deprivation status based on their experiences across the component indicators. The deprivation score for each person was determined by calculating a weighted sum of the deprivations encountered. This score ranges from zero to one, and an individual is classified as poor if their deprivation exceeds 33% of the weighted indicators; otherwise, they are considered non-poor.

### Data processing and analysis

To ensure data quality and consistency, we conducted data cleaning, recoding, variable generation, and labeling using Stata version 17.0. To address the unequal probability of selection between geographically defined strata and non-responses, we used sample weights. Frequencies and percentages were calculated for categorical variables to describe the characteristics of the study population based on the multidimensional poverty index and inadequate MDD.

During analysis, the survey design was taken into account and declared. The Pearson chi-square test was used to examine whether the disparity in the multidimensional poverty index and inadequate MDD was statistically significant. To explain the multidimensional poor and non-poor disparities in inadequate MDD among children aged 6–23 months, multivariate decomposition, which is the extension of Blinder-Oaxaca decomposition analysis for the non-linear dependent variable (30), was used.

#### Decomposition analysis

Multivariate decomposition was used. Primarily, multivariate decomposition is intended for non-linear decomposition and provides convenient methods for addressing path dependency and overcoming the identification problem associated with the selection of a reference category in the case of Blinder-Oaxaca decomposition analysis. This approach automatically determines the high-outcome group as the comparison group, using the low-outcome group as the reference. A detailed multivariate decomposition analysis was conducted to investigate the disparity in inadequate MDD between non-poor and poor children aged 6–23 months in SSA. In this study, Y is considered the outcome variable (inadequate MDD), the multidimensional poverty index (MPI) serves as the decomposing variable (non-poor and poor), X represents the explanatory variable, and ß denotes the coefficient of the explanatory variable. Inadequate MDD for multidimensional non-poor and poor groups is represented as follows (Equation 1).


(1) $${\bar{Y}}_{1}-{\bar{Y}}_{0}\;=\Delta x\beta_{0}+\Delta x\beta_{1}$$

where $Y{ˉ}_{1}$ and $Y{ˉ}_{0}$ represent the mean outcome for the non-poor group and the mean outcome for the poor group, respectively. $\Delta x\beta_{1}$ is the explained component (E), representing the difference in outcomes due to differences in the levels of determinants (covariates) between the two groups, and $\Delta x\beta_{0}$ is the unexplained component (C), representing the difference in outcomes due to differences in the effects of the determinants (coefficients) between the two groups. Finally, $\beta_{1}$ and $\beta_{0}$ are the coefficients for the non-poor group and the coefficients for the poor group, respectively.

Therefore, the gap in average inadequate MDD is assumed to develop from a gap in endowments (E) and a gap in coefficients (C). A *p*-value of less than 0.05 was considered statistically significant.

#### Ethical approval

No ethical approval was required for this study, as we utilized the DHS data, which had been anonymized before public release. The DHS datasets used in this study are freely accessible. We obtained an authorization letter to download the DHS dataset from the Central Statistical Agency (CSA) through the DHS Program website at https://dhsprogram.com/. The dataset and all methodologies employed in this study adhered to the guidelines established in the Declaration of Helsinki and followed the DHS research protocols.

## Results

### The sociodemographic characteristics and health service utilization of respondents with MDD and MPI

Table 2 indicates that maternal age has a modest impact on achieving adequate MDD, particularly among mothers in the 40–44 age group, which represents 4,917 (13.18%) of the total. In contrast, early maternal age (15–19 years) is linked to inadequate MDD, which represents 109,139 (92.34%) of the total. The influence of maternal age on the Multidimensional Poverty Index (MPI) differs from its effect on MDD. Mothers in the 15–19 age group falls into the multidimensional non-poor category, accounting for 10,285 (87.03%), while those in the 30–34 age group is classified as multidimensional poor, making up 20,892 (24.02%) of the total.

**Table 2:** Sociodemographic characteristics of the respondents in the study area with MDD and MPI.

		MDD		MPI	
Variables	Category	Adequate	Inadequate	Non-poor	Poor
Maternal age	15–19 years	906 (7.66)	10,913(92.34)	10,285(87.03)	1,533(12.97)
	20–24 years	4,798(10.35)	41,565(89.65)	39,624(85.46)	6,739(14.54)
	25–29 years	8,712(10.64)	73,149(89.36)	64,946(79.34)	16,915(20.66)
	30–34 years	9,589(11.03)	77,380(88.97)	66,077(75.98)	20,892(24.02)
	35–39 years	8,598(10.93)	70,050(89.07)	59,864(76.12)	18,785(23.88)
	40–44 years	4,917(13.18)	32,387(86.82)	28,784(77.16)	8,519(22.84)
	45–49 years	1,054(11.1)	8,445(88.9)	7,286(76.7)	2,214(23.3)
Sub-Saharan African region (SSA)	Central Africa (CA)	2,944(11.76)	22,088(88.24)	20,343(81.27)	4,689 (18.73)
	West Africa (WA)	15,165(9.92)	137,662(90.08)	127,897(83.69)	24,930 (16.3)
	East Africa (EA)	8,112(11.16)	64,556(88.84)	48,248(66.4)	24,419(33.6)
	South Africa (SA)	12,354(12.12)	89,583(87.88)	80,378(78.85)	21,558(21.15)
Residence	Urban	15,084(14.05)	92,314(85.95)	88,363(82.28)	19,035(17.72)
	Rural	23,490(9.59)	221,575(90.41)	188,503(76.92)	56,562(23.08)
Highest educational level	No education	14,745(8.5)	158,733(91.5)	141,176(81.38)	32,303(18.62)
	Primary education	12,109 (11.4)	94,065(88.6)	78,715(74.14)	27,459(25.86)
	Secondary education	9,673(15.27)	53,685(84.73)	49,825(78.64)	13,533(21.36)
	Higher education	2048(21.66)	7,405(78.34)	7,150(75.64)	2,303(24.36)
Sex of household head	Male	32,705 (11.13)	261,223(88.87)	231,774(78.85)	62,154(21.15)
	Female	5,870(10.03)	52,665(89.97%)	45,092(77.03)	13,444(22.97)
Wealth index	Poorest	6,678 (7.1)	87,420(92.9)	68,857(73.18)	25,241(26.82)
	Poorer	7,457 (9.24)	73,207(90.76)	63,262(78.43)	17,402(21.57)
	Middle	7,359 (10.34)	63,813(89.66)	57,349(80.58)	13,823(19.42)
	Richer	8,300(13.78)	51,936(86.22)	49,854(82.76)	10,382(17.24)
	Richest	8,781(18.97)	37,514(81.03)	37,545(81.1)	8,750(18.9)
Husband/partner’s education level	No education	13,111(8.61)	139,223(91.39)	125,400(82.32)	26,934(17.68)
	Primary education	9,369(11.79)	70,086(88.21)	63,763(80.25)	15,692(19.75)
	Secondary education	9,027(14.94)	51,415(85.06)	50,055(82.81)	10,388(17.19)
	High education	3,366 (18.84)	14,497 (81.16)	15,044 (84.22)	2,819 (15.78)
Husband’s/partner’s occupation	Unemployed	13,556 (11.32)	106,250 (88.68)	99,179 (82.78)	20,628 (17.2)
	Employed	21,518(11.21)	170,507(88.79)	156,530(81.52)	35,495(18.48)
Respondents’ occupation	Unemployed	15,997 (10.61)	134,848 (89.39)	124,266(82.38)	26,579(17.62)
	Employed	20,647 (11.83)	153,878 (88.17)	142,129(81.44)	32,396(18.56)
Types of birth	Single	37,543(10.97)	304,716(89.03)	269,879(78.85)	72,380(21.15)
	Multiple	1,031(10.11)	9,173(89.89)	6,987(68.47)	3,217(31.53)
Sex of child	Male	19,709(11.0)	159,438(89)	139,980(78.14)	39,167(21.86)
	Female	18,866(10.89)	154,451(89.11)	136,886(78.98)	36,430(21.02)
Preceding birth interval	Less than 2 year	6,487(10.38)	56,000(89.62)	45,689(73.12)	16,798(26.88)
	2 year	936(10.65)	7,852(89.35)	6,726(76.53)	2062(23.47)
	Greater than 2 yr	19,317(10.8)	159,537(89.2)	147,264(82.34)	31,590(17.66)
Antenatal check	No	84(10.93)	686(89.07)	705(91.52)	65(8.48)
	Yes	10,973(12.08)	79,894(87.92)	84,636(93.14)	6,231(6.86)
Place of delivery	Home	3,678(8.03)	42,125(91.97)	37,398(81.65)	8,405(18.35)
	Health facility	13,756(12.39)	97,239(87.61)	102,298(92.16)	8,697(7.84)
Current marital status	Non-union	2,990(10.46)	25,601(89.54)	22,550(78.87)	6,042(21.13)
	Union	35,585(10.99)	288,287(89.01)	254,317(78.52)	69,555(21.48)

Geographically, East Africa has a higher concentration of multidimensional poor individuals, totaling 24,419 (33.16%), followed by South Africa with 21,558 (21.15%). Additionally, disparities between urban and rural areas significantly impact both MDD and MPI. As shown in the table, urban areas positively contribute to MDD adequacy, with 15,084 (14.05%), and have a substantial number of multidimensional non-poor individuals at 88,363 (82.28%). Conversely, rural areas experience higher levels of MDD inadequacy at 221,575 (90.41%), with multidimensional poor individuals numbering 56,565 (23.08%). Maternal education also influences MDD inadequacy and MPI; mothers with no formal education significantly contribute to MDD inadequacy, totaling 158,733 (91.5%). In terms of multidimensional poverty, those with primary education represent a notable share at 27,459 (25.86%), followed by mothers with higher education at 2,303 (24.36%). Furthermore, the gender of the household head affects MDD inadequacy and MPI outcomes in the region. The data reveals that MDD inadequacy is prevalent among women-headed households, accounting for 52,665 (89.97%), while the total number of multidimensional poor individuals in this category is 13,444 (22.97%) (Table 2).

### Prevalence of inadequate MDD in SSA

The overall prevalence of inadequate MDD among children in SSA was 89.05% (95% CI, 89.2–89.95). Within multidimensional poor households, the prevalence of inadequate MDD among children was 91.8%, while the prevalence of multidimensional non-poor households in SSA was 88.2%. The highest prevalence of inadequate MDD was observed in Burkina Faso (detailed prevalence for each country is provided in Table 1).

### Multidimensional poor versus non-poor disparity of inadequate MDD in SSA

**Figure 1:**
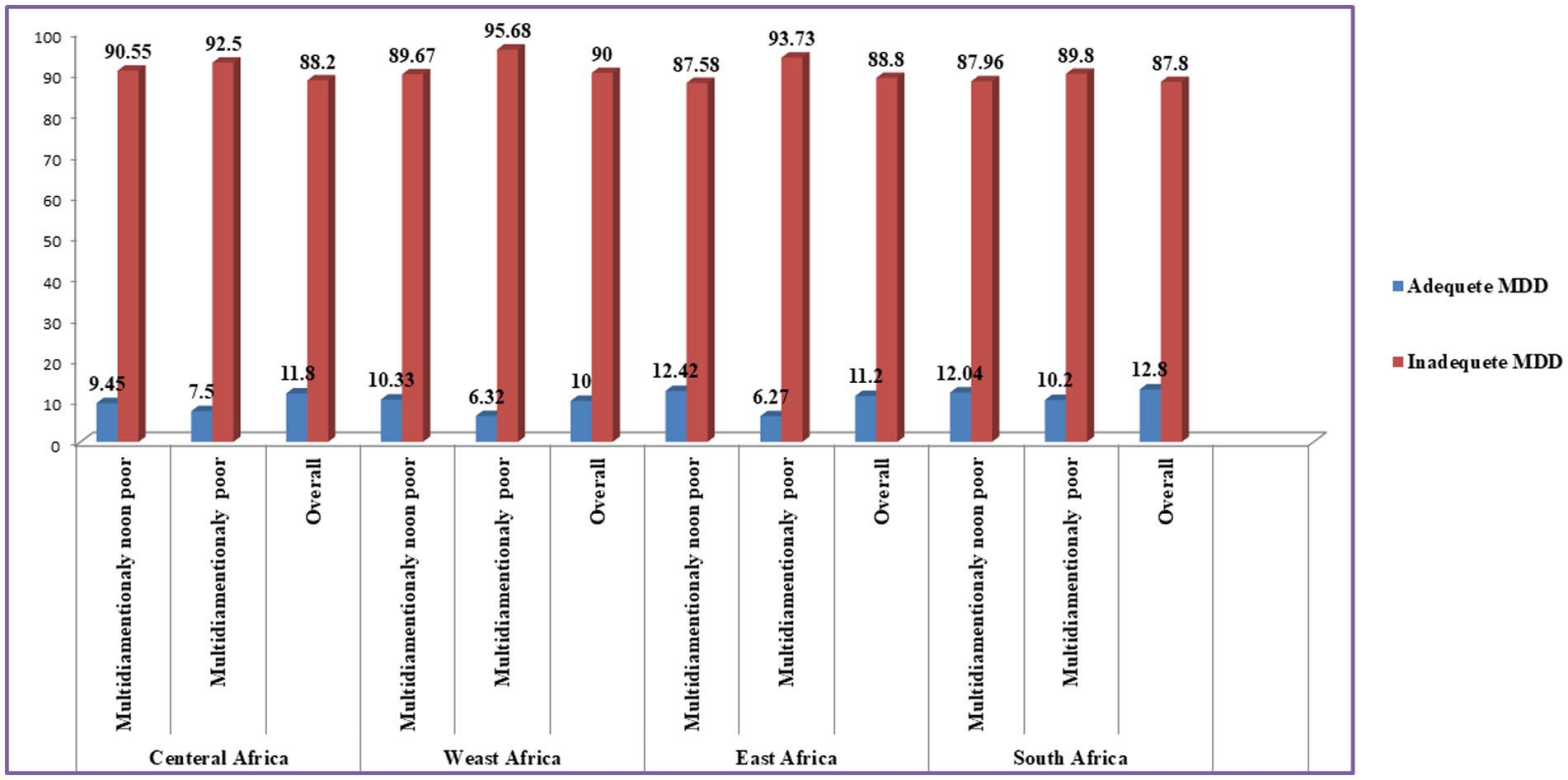
Proportion of inadequate MDD in Sub-Saharan Africa stratified by multidimensional poverty index.

Figure 1 depicts the differences in inadequate minimum dietary diversity (MDD) between multidimensional poor and non-poor populations in Sub-Saharan Africa (SSA). In this region, the prevalence of inadequate MDD among children was highest in Central Africa (90.55, 95% CI: 87.5–88.9), followed by West Africa (90, 95% CI: 89.9–90.25), East Africa (88.8, 95% CI: 88.5–89.2), and South Africa (87.8, 95% CI, 87.6–88.1). The disparity in inadequate MDD between the multidimensional poor and non-poor was most pronounced in East Africa (6.15%) and least pronounced in Central Africa (1.95%) (Figure 1). This difference was found to be highly statistically significant (chi-square, *p* = 0.000).

### Multivariate decomposition of analysis of inadequate MDD

A decomposition analysis model used in this study accounted for differences in characteristics (compositional factors) and differences due to the effects of factors (coefficients).

### Difference due to characters (endowment)

Approximately 55.55% of the overall inadequate MDD difference was due to differences in characteristics. Among the demographic factors, maternal age, the educational and occupational status of both parents, the sex and age of the household head, wealth index, place of residence, the number of living children, and regions of SSA significantly contributed to the disparity in inadequate MDD among children in SSA (Table 2). The negative coefficient for each independent variable at a *p*-value of <0.05 indicates that the characteristics associated with inadequate MDD help narrow the gap in inadequate MDD between multidimensional poor and non-poor children aged 6–23 months in SSA. For instance, the maternal age groups of “40–44” and “45–49” years show statistically significant negative coefficients, reducing the gap in inadequate MDD between multidimensional poor and non-poor children by 10.2 and 6.4%, respectively, compared to the reference category of “15–19” years old. In practical terms, if the maternal age in multidimensional poor households were to increase to the age groups of “40–44” and “45–49,” the gap in the prevalence of inadequate MDD between multidimensional poor and non-poor children in SSA would decrease by 10.2 and 6.4%, respectively.

The gaps in inadequate MDD between multidimensional poor and non-poor children in SSA narrowed by 0.2% when the educational status of the father was primary education. However, these gaps increased by 3.43 and 2.24% when the father’s educational level was secondary education and higher education, respectively, compared to no education. The gaps in inadequate MDD among children in SSA decreased by 4.2% when the father’s occupational status indicated being employed compared to children in multidimensional poor households. The positive coefficient for each independent variable, significant at a *p*-value of <0.05, indicates that the factors associated with inadequate MDD contribute to the widening gap of inadequate MDD between multidimensional poor and non-poor children aged 6–23 months in SSA. For instance, the coefficient for the category “>7 family members” is 0.001 with a *p*-value of 0, indicating that an increase in household size in multidimensional poor households raises the gap in inadequate MDD prevalence between multidimensional poor and non-poor children by 3.05%. Factors such as rural residence, female household head, and the increasing age of the household head have contributed to the widening gap in inadequate MDD between multidimensional poor and non-poor children aged 6–23 months in SSA (Table 3).

**Table 3:** Multivariate decomposition analysis of multidimensional poor-non-poor disparity in minimum dietary diversity among children aged 6–23 months in Sub-Saharan Africa using recent Demographic and Health Survey.

Decompose	Coef.	*P*-value	Percent		
Endowment	−0.1314	0	−55		
Coefficient	0.039	0	155		
Row difference	0.026	0			

	Due to the difference in characteristics (E)			Due to the difference in coefficients (C)		
MDD	Coef.	*P*-value	Percent	Coef.	*P*-value	Percent
Maternal age						
15–19 years	Refer.					
20–24 years	0.002	0.5	5.61	0.001	0.7	3.28
25–29 years	0.001	0.8	3.7	0.007	0.3	27.26
30–34 years	−0.001	0.12	−4.8	0.001	0.9	4.98
35–39 years	−0.004	0.14	−16.45	0.001	0.9	4.97
40–44 years	−0.003	0.03	−10.2	0.001	0.89	1.76
45–49 years	−0.002	0.002	−6.4	−0.001	0.38	−3.11
Occupational status						
Unemployed	Refer.					
Employed	−0.001	0.006	−0.2	0.002	0.191	8.74
Household member						
<5	Refer.					
5–7	0.002	0.34	0.61	−0.002	0.401	−7.2
>7 members	−0.001	0.09	−0.53	−0.001	0.438	−7.72
Number of children						
<4	Refer.					
≥4	0.001	0	3.05	−0.001	0	−3.98
Educational status of father						
No education	Refer.					
Primary	−0.001	0	−4.2	−0.003	0.001	−10.9
Secondary	0.001	0	3.43	−0.003	0	−13.4
Higher	0.001	0.001	2.24	−0.001	0.23	−1.8
Partner’s occupation						
Unemployed	Refer.					
Employed	−0.001	0	−2	−0.0039	0.048	−15.2
Residence						
Urban	Refer.					
Rural	0.002	0.005	5.22	0.008	0.004	31.8
Educational status of mother						
No education	Refer.					
Primary	−0.007	0	−27	−0.007	0	−28.7
Secondary	−0.003	0	−9.3	−0.005	0	−18.7
Higher	−0.001	0	0.1	−0.001	0	−2.7
Sex of household head						
Male	Reference					
Female	0.001	0	3.85	0.002	0.003	6.5
Age of household head	0.001	0.015	1.1	0.004	0.586	14.56
Wealth index						
Poorest	Reference	.	0	0	.	0
Poorer	−0.001	0.001	−0.7	−0.0001	0.921	−0.38
Middle	0.001	0.002	2.4	0.002	0.067	6.7
Richer	0.001	0.18	2.17	0.006	0	22
Richest	0.002	0	7.67	0.003	0	13.1
Sex of the child						
Male	Reference					
Female	0.001	0.6	0.1	−0.002	0.16	−7.66
Types of birth						
Single	Reference					
Multiple	0.0004	0.22	1.41	0.001	0.086	1.42
Preceding birth interval						
≤2	Reference					
>2 year	0.001	0.6	1.31	0.002	0.001	10.3
SSA region						
Central Africa	Reference					
West Africa	−0.002	0.005	−6.6	0.007	0.005	29.2
East Africa	−0.001	0.4	−0.46	0.001	0.307	4.44
South Africa	−0.001	0.08	−4.72	0.001	0.706	2.59

### Difference due to the effect of characters (coefficient)

After controlling for individual and compositional factors, 155.87% of the inadequate MDD difference was due to the differences in the effects of characteristics. The educational status of both parents and the father’s occupational status helped narrow the inadequate MDD gap between multidimensional poor and non-poor children, while sex of the household head, household wealth index, preceding birth interval, place of residence, and African regions contributed to widening the inadequate MDD gap between multidimensional poor and non-poor children (Table 2). For instance, the positive coefficient for place of residence (0.008) showed that children from rural areas contribute to a 31.8% increase in the inadequate MDD gap between multidimensional poor and non-poor children. Conversely, the father’s employment status has a statistically significant negative coefficient, with a percentage contribution of −15.2%. This negative contribution suggests that father’s employment is associated with a reduction in the inadequate MDD gap between multidimensional poor and non-poor children (Table 3).

### Detailed multivariate decomposition analysis in each region of Africa

#### Difference due to characters (endowment)

In the detailed multivariate decomposition, the study revealed that the explained differences due to changes in compositional characteristics were 227.85% for Central Africa, 217.6% for East Africa, and 15.6% for South Africa. Among these compositional factors, multiple births (17.14%) and rural residence (97.2%) significantly contributed to the widening of gaps, while the educational status of both parents, the sex of the household head, and the preceding birth interval played a role in narrowing the inadequate MDD gap between the multidimensional poor and non-poor children (Table 4). In West Africa, the compositional and demographic factors (explained component) were insignificant (Table 4). Regarding the compositional factors in East Africa, the educational status of both parents contributed to narrowing the gap, while the age of the household head and the size of the household significantly impacted the widening of the inadequate MDD gap between multidimensional poor and non-poor children (Table 5). In South Africa, compositional factors such as maternal age, the educational and working status of the mother, the age and sex of the household head, the number of live children, and place of residence made a significant contribution to the disparity in inadequate MDD among children (Table 5).

**Table 4:** Detailed multivariate decomposition analysis of multidimensional poor-non-poor disparity in minimum dietary diversity among children aged 6–23 months in Central Africa and West Africa using recent Demographic and Health Survey.

	Central Africa				West Africa			
Decompose	Coef.	Std. Err.	*P*-value	Pct.	Coef.	Std. Err.	*P*-value	Pct.
Endowment	−0.05	0.004	0	227.9	0.001	0.001	0.426	1.81
Coefficient	0.024	0.007	0.001	−127.9	0.043	0.002	0	98.19
Row difference	−0.02	0.006	0.002		0.044	0.002	0	

	Endowment				Endowment			
Decompose	Coef.	Std. Err.	*P*-value	Pct.	Coef.	Std. Err.	*P*-value	Pct.
Maternal age								
15–19 years	Reference							
20–24 years	−0.022	0.012	0.068	114.5	−0.003	0.006	0.661	−5.600
25–29 years	−0.043	0.025	0.092	227.8	0.001	0.001	0.657	−0.960
30–34 years	0.003	0.003	0.325	−14.4	0.001	0.002	0.653	1.560
35–39 years	0.020	0.022	0.361	−104.7	0.001	0.002	0.652	1.590
40–44 years	−0.002	0.014	0.876	11.5	0.001	0.000	0.654	0.490
45–49 years	0.001	0.005	0.936	2.0	0.001	0.001	0.655	1.020
Occupational status								
Unemployed	Reference							
Employed	0.001	0.000	0.474	−0.450	0.001	0.000	0.678	0.1
Household member								
<5	Reference							
5–7 members	0.001	0.000	0.008	−5.990	−0.001	0.000	0.916	−0.030
>7	0.001	0.00	0.00	−16.98	0.001	0.00	0.68	−0.94
Number of children								
≤4	Reference							
>4	0.001	0.000	0.968	0.02	0.001	0.001	0.663	1.360
Edu. status of the father								
No education	Reference							
Primary	−0.01	0.00	0.04	26.05	0.001	0.00	0.68	−0.18
Secondary	0.001	0.00	0.38	−6.94	0.001	0.00	0.68	0.00
Higher	−0.01	0.00	0.02	30.63	0.001	0.00	0.67	0.07
Partner occupation								
Unemployed	Reference							
Employed	0.0001	0	0.61	1.7	0.0001	0	0.67	0.41
Residence								
Urban	Reference							
Rural	0.018	0.009	0.031	−97.19	0.0001	0.000	0.749	−0.240
Educational status of mother								
No education	Reference							
Primary	0.001	0.00	0.30	18.91	0.001	0.00	0.65	4.34
Secondary	−0.03	0.01	0.00	147.88	0.001	0.00	0.65	3.71
Higher	0.01	0.00	0.00	−64.14	0.001	0.00	0.65	1.09
Sex of household head								
Male	Reference							
Female	0.001	0.00	0.03	−5.16	0.001	0.00	0.67	−0.23
Age of household head	0.001	0.00	0.62	2.34	0.001	0.00	0.70	0.13
Wealth index								
Poorest	Reference							
Poorer	0.001	0.00	0.12	−15.22	0.001	0.00	0.68	−0.15
Middle	0.001	0.00	0.35	12.19	0.001	0.00	0.68	−1.10
Richer	0.001	0.01	0.45	−20.12	0.001	0.00	0.68	−1.58
Richest	0.001	0.00	0.13	19.96	0.001	0.00	0.68	−1.55
Sex of the child								
Male	Reference							
Female	0.001	0.00	0.53	0.53	0.001	0.00	0.68	−0.27
Types of birth								
Single	Reference							
Multiple	0.001	0.00	0.11	17.14	0.001	0.00	0.69	−1.10
Precede birth interval								
≤2	Reference							
>2 year	0.01	0.00	0.02	−53.99				

	Due to coefficient (C)				Due to coefficient (C)			
Decompose	Coef.	Std. Err.	*P*-value	Pct.	Coef.	Std. Err.	*P*-value	Pct.
Maternal age								
15–19 years	Reference							
20–24 years	0.01	0.00	0.07	−44.66	−0.01	0.00	0.03	−14.14
25–29 years	0.02	0.01	0.05	−117.6	0.001	0.01	0.60	−6.68
30–34 years	0.01	0.01	0.38	−63.89	0.001	0.01	0.71	−4.86
35–39 years	0.01	0.01	0.40	−51.22	0.001	0.01	0.70	−4.40
40–44 years	0.001	0.01	0.46	−22.20	0.001	0.00	0.83	−1.13
45–49 years	0.001	0.00	0.92	0.60	0.001	0.00	0.04	−3.51
Occupational status								
Unemployed	Reference							
Employed	0.01	0.00	0.045	−40.49	0.001	0.00	0.15	8.50
Household member								
<5	Reference							
5–7 members	0.02	0.00	0.001	−83.92	0.001	0.00	0.24	−5.84
>7	0.02	0.00	0	−92.93	−0.01	0.00	0.03	−16.53
Number of children								
≤4	Reference							
>4	0.001	0.00	0.23	4.67	0.001	0.00	0.00	7.87
Educational status of father								
No education	Reference							
Primary	0.001	0.00	0.96	0.37	0.001	0.00	0.12	2.95
Secondary	0.001	0.00	0.73	−5.02	0.001	0.00	0.44	1.85
Higher	0.01	0.00	0.001	−31.72	0.001	0.00	0.001	4.01
Partner occupation								
Unemployed	Reference							
Employed	−0.01	0.01	0.11	42.27	0.001	0.00	0.76	2.05
Residence								
Urban	Reference							
Rural	0.01	0.00	0.14	−27.27	0.001	0.00	0.71	2.77
Educational status of mother								
No education	Reference							
Primary	0.001	0.00	0.63	5.73	−0.01	0.00	0	−14.07
Secondary	0.001	0.00	0.24	23.78	0.001	0.00	0	−17.32
Higher	−0.01	0.00	0	25.65	0.001	0.00	0	−2.11
Sex of household head								
Male	Reference							
Female	0.001	0.00	0.13	−11.66	0.001	0.00	0.004	4.84
Age of household head	0.01	0.02	0.62	−47.72	−0.02	0.01	0.14	−27.16
Wealth index								
Poorest	Reference							
Poorer	−0.01	0.00	0.012*	31.47	0.001	0.00	0.88	0.47
Middle	0.001	0.00	0.41	−10.85	0.001	0.00	0.41	2.48
Richer	0.001	0.00	0.73	3.93	0.001	0.00	0.07	5.22
Richest	0.01	0.00	0.018	−29.29	0.001	0.00	0.87	−0.39
Sex of the child								
Male	Reference							
Female	−0.007	0.004	0.047	38.320	−0.004	0.002	0.029	−9.630
Types of birth								
Single	Reference							
Multiple	−0.001	0.000	0.204	3.340	0.001	0.000	0.02	1.420
Precede birth interval								
≤2	Reference							
>2 year	0.01	0.00	0.00	−65.21	0.08	0.02	0.001	182.5

**Table 5:** Detailed multivariate decomposition analysis of multidimensional poor-non-poor disparity in minimum dietary diversity among children aged 6–23 months in East Africa and South Africa using recent Demographic and Health Survey.

	East Africa				South Africa			
Decompose	Coef.	Std. Err.	*P*-value	Percent	Coef.	Std. Err.	*P*-value	Percent
Endowment	−0.06	0.00	0.00	217.60	−0.01	0.00	0.00	−15.92
Coefficient	0.04	0.01	0.00	−117.6	0.04	0.00	0.00	115.92
Row difference	−0.03	0.00	0.00		0.03	0.00	0.00	

Endowment difference in characteristics (E)								
Decompose	Coef.	Std. Err.	*P*-value	Percent	Coef.	Std. Err.	*P*-value	Percent
Maternal age								
15–19 years	Reference							
20–24 years	−0.03	0.02	0.00	120.82	0.001	0.00	0.31	−2.31
25–29 years	−0.07	0.02	0.00	243.06	0.001	0.00	0.66	−1.10
30–34 years	−0.01	0.00	0.02	39.60	0.001	0.00	0.50	−0.97
35–39 years	0.04	0.02	0.01	−156.7	0.001	0.00	0.32	−2.22
40–44 years	0.01	0.01	0.04	−44.68	0.001	0.00	0.11	−1.35
45–49 years	0.001	0.00	.	0.00	0.001	0.00	0.042	−1.50
Occupational status								
Unemployed	Reference							
Employed	0.001	0.00	0.72	−0.32	0.001	0.00	0.016	−0.27
Household member								
<5	Reference							
5–7 members	0.001	0.00	0.65	0.69	0.001	0.00	0.45	0.15
>7	0.03	0.01	0.03	−93.61	0.001	0.00	0.46	0.03
Number of children								
≤4	Reference							
>4	0.001	0.01	0.94	−2.50	0.001	0.00	0.012	−4.40
Edu. status of father								
No education	Reference							
Primary	0.001	0.00	0.09	2.94	−0.01	0.00	0.00	−1.88
Secondary	0.001	0.00	0.83	−1.51	0.001	0.00	0.00	1.97
Higher	0.001	0.00	0.18	−12.73	0.001	0.00	0.00	1.04
Partner occupation								
Unemployed	Reference							
Employed	0.001	0.00	0.037	1.10	0.001	0.00	0.02	−0.18
Residence								
Urban	Reference							
Rural	−0.01	0.00	0.13	20.05	0.001	0.00	0.014	1.29
Edu. status of mother (no)								
No education	Reference							
Primary	−0.04	0.01	0	149.8	0.001	0.00	0.021	−4.16
Secondary	−0.01	0.00	0	22.8	0.001	0.00	0.012	−0.85
Higher	−0.01	0.00	0	19.5	0.001	0.00	0.3	0.02
Sex of household head								
Male	Reference							
Female	0.005	0.003	0.123	−17.23	0.0001	0.000	0.003	1.140
Age of household head	0.030	0.014	0.037	−107.9	0.0001	0.000	0.043	0.490
Wealth index								
Poorest	Reference							
Poorer	0.001	0.00	0.90	0.27	0.001	0.00	0.04	−0.29
Middle	0.001	0.00	0.68	−1.65	0.001	0.00	0.26	0.14
Richer	0.001	0.00	0.79	4.21	0.001	0.00	0.14	−0.59
Richest	0.01	0.01	0.05	−36.66	0.001	0.00	0.006	1.18
Sex of the child								
Male	Reference							
Female	0.001	0.00	0.28	3.47	0.001	0.00	0.13	−0.09
Types of birth								
Single					Reference			
Multiple					0.001	0.00	0.10	−0.29
Precede birth interval								
≤2	Reference							
>2 year	−0.01	0.01	0.04	49.57	0.001	0.00	0.09	−1.05

Decompose	Due to coefficient (C)				Due to coefficient (C)			
Maternal age								
15–19 years	Reference							
20–24 years	0.01	0.00	0.001	−35.00	0.01	0.00	0.032	20.61
25–29 years	0.02	0.01	0	−88.38	0.01	0.01	0.072	33.88
30–34 years	0.02	0.01	0.009	−79.78	0.01	0.01	0.20	26.15
35–39 years	0.03	0.01	0.003	−96.67	0.01	0.01	0.27	21.33
40–44 years	0.02	0.00	0.001	−60.27	0.001	0.00	0.40	8.34
45–49 years	0.001	0.00	0.008	−10.63	0.001	0.00	0.91	0.23
Occupational status								
Unemployed	Reference							
Employed	0.001	0.00	0.20	−16.23	0.001	0.00	0.41	−5.17
Household member								
<5	Reference							
5–7 members	−0.01	0.01	0.39	21.70	0.001	0.00	0.47	−7.05
>7	0.01	0.00	0.21	−19.95	−0.01	0.00	0.02	−25.04
Number of children								
≤4					Reference			
>4					0.01	0.003	0.005	27.890
Edu. status of father								
No education	Reference							
Primary	−0.01	0.01	0.25	30.32	−0.01	0.00	0	−18.47
Secondary	0.001	0.00	0.81	1.98	−0.01	0.00	0	−21.52
Higher	0.001	0.00	0.73	1.38	0.001	0.00	0	−5.44
Partner occupation								
Unemployed	Reference							
Employed	−0.01	0.00	0.044	25.66	0.001	0.00	0.39	−6.79
Residence								
Urban	Reference							
Rural	0.01	0.01	0.29	−36.74	0.01	0.00	0.27	15.06
Edu. status of mother (no)								
No education	Reference							
Primary	−0.03	0.01	0	120.3	0.001	0.00	0.28	−5.63
Secondary	−0.01	0.00	0.006	21.95	0.001	0.00	0.026	−6.25
Higher	0.001	0.00	0.001	8.56	0.001	0.00	0.22	0.64
Sex of household head								
Male	Reference							
Female	0.0001	0.001	0.964	−0.220	0.0001	0.001	0.588	1.270
Age of household head	0.053	0.02	0.012	−195	0.013	0.009	0.145	38.76
Wealth index								
Poorest	Reference							
Poorer	0.001	0.002	0.661	−3.6	0.001	0.001	0.466	2.710
Middle	0.004	0.002	0.058	−14.7	0.001	0.001	0.652	1.590
Richer	0.009	0.003	0*	−33.0	0.007	0.001	0	19.410
Richest	0.007	0.002	0.005	−24.4	0.004	0.001	0.001	11.550
Sex of the child								
Male	Reference							
Female	0.001	0.00	0.20	15.74	0.001	0.00	0.44	4.15
Types of birth								
Single	Reference							
Multiple	0.001	0.00	0.15	1.16	0.001	0.00	0.78	−0.20
Precede birth interval								
≤2	Reference							
>2 year	0.001	0.00	0.25	7.43	0.001	0.00	0.78	0.98

The numbers in the table are rounded to the nearest three decimal points, which means that the zero point does not exactly indicate zero value.

#### Difference due to the effect of characters (coefficient)

The differences in the effects of characteristics indicated that 127.85% of the disparity occurred in Central Africa, 98.19% in West Africa, 117.6% in East Africa, and 115.92% in South Africa.

After controlling for individual and compositional factors, 127.85% of the disparity in inadequate MDD was due to differences in the effects of characteristics.

A significant difference in inadequate MDD, driven by differences in characteristic effects was associated with the educational status of both parents, the father’s occupational status, household size, and the household wealth index in Central Africa (Table 4).

In West Africa, 98.19% of the disparity in inadequate MDD was due to differences in the effects of various characteristics. A significant difference in inadequate MDD due to differences in the effects of characteristics (coefficient) was associated with the educational status of both parents, the sex of the household head, the number of living children, types of birth, and the preceding birth interval (Table 3).

After controlling for individual and compositional factors, 117.6% of the disparity in inadequate MDD remained unexplained due to variations in the effects of characteristics across East Africa. A significant difference in inadequate MDD due to differences in the effects of characteristics (coefficient) difference was observed. Key contributors to this difference included the mother’s educational status, the father’s occupational status, the age of the household head, and the wealth index (Table 5).

After controlling for individual and compositional factors in South Africa, 115.92% of the inadequate MDD difference remained unexplained due to the varying effects of characteristics. A significant difference in inadequate MDD due to these varying effects (coefficient) was associated with the educational status of the father, the number of children, the age of the household head, and the wealth index (Table 5).

## Discussion

The study aimed to assess disparities in inadequate minimum dietary diversity (MDD) among children aged 6–23 months in Sub-Saharan Africa (SSA) between multidimensional poor and non-poor households. The findings reveal a high prevalence of inadequate MDD across SSA, with significant disparities between poor and non-poor households. The overall prevalence of inadequate MDD was 89.05%, with the highest prevalence in Central Africa (90.55%) and the lowest in South Africa (87.8%). The prevalence of inadequate MDD in SSA exceeds that of other regions, such as South Asia and Southeast Asia, where similar studies have been conducted (26, 31, 32), aligning with previous study findings (17, 33). This disparity could be attributed to limited access to child health services (34) and may be influenced by rapid population growth, socioeconomic disparities, drought, and various other natural and man-made factors that impact nutrition in SSA.

The disparity in inadequate MDD between multidimensional poor and non-poor households was most pronounced in East Africa (6.15%) and least pronounced in Central Africa (1.95%), which was higher than the figures from previous studies conducted in Bangladesh, India, and other Asian countries (32, 35). This variation may be due to environmental changes, socioeconomic variations, and differences in healthcare utilization. These findings highlight the urgent need for targeted interventions to address the nutritional needs of children in SSA, particularly in poor households.

The disparity in inadequate MDD between multidimensional poor and non-poor households is particularly concerning. The findings indicate that children in poor households are significantly more likely to experience inadequate MDD compared to their non-poor counterparts. This disparity is most pronounced in East Africa, where the difference between poor and non-poor households is high. This finding was in line with previous study findings (27, 36), which suggests that poverty is a major driver of inadequate dietary diversity in the region, and efforts to reduce poverty could have a significant impact on improving children’s nutritional outcomes.

The multivariate decomposition analysis revealed that several factors contribute to the disparity in inadequate MDD between poor and non-poor households. These factors include maternal education, paternal education, household wealth, residence, family size, and the number of children. The findings of this study are consistent with previous research conducted in SSA and other low- and middle-income countries. For example, studies in Ethiopia, Bangladesh, and India have also identified poverty, maternal education, and household wealth as significant determinants of inadequate MDD among children (26, 27, 32).

Maternal education was found to be a key factor in narrowing the gap in inadequate MDD between poor and non-poor households. A study conducted in India and Bangladesh utilized socioeconomic inequality decomposition analysis to support this finding (26, 27, 32). The potential reason is that educated mothers are more likely to address the nutritional needs and are better equipped to make informed decisions regarding their children’s diets.

Household wealth also plays a significant role in dietary diversity. Poor households are more likely to face economic constraints that limit their ability to purchase diverse foods, which is in line with other study findings (14, 26, 32). This finding is particularly true in rural areas, where access to markets and diverse food options is often limited.

The study found that rural residence was associated with a higher prevalence of inadequate MDD, consistent with previous research (37, 38). This underscores that children in rural areas often face greater challenges in accessing diverse and nutritious foods, which can be attributed to factors such as limited economic resources, fewer food options, and reduced health services.

The finding indicated that poverty, lower education levels, and rural residency contribute to the widening gap of inadequate MDD between multidimensional poor and non-poor households in SSA compared to their counterparts, which is consistent with other study findings (26, 32, 39, 40). One possible reason is that individuals in rural areas who are less educated and economically disadvantaged may be less likely to consume diversified foods due to economic constraints. For example, they may sell eggs to purchase other food items that are less expensive to alleviate their financial difficulties.

Family size and the number of children were also found to contribute to the disparity in inadequate MDD, which is in line with findings from other studies (26, 32, 39, 40). This disparity may be because larger households with more children face greater challenges in providing adequate dietary diversity due to limited resources. This situation is particularly evident in poorer households, where resources are already stretched thin.

### Indications for policy and interventions

The findings of this study have significant implications for policies and interventions aimed at improving dietary diversity among children in SSA. Efforts should be made to reduce poverty and improve economic opportunities, particularly for women. This finding could include programs that provide financial support, improve access to education, and promote economic activities for women. Reducing poverty would not only improve dietary diversity but also yield broader benefits for child health and development. Finally, the study highlights the need for targeted interventions in regions with the highest disparities in inadequate MDD, such as East Africa. These interventions should be tailored to the specific needs of the region and address the underlying causes of poverty and food insecurity.

### Strengths and limitations

This study’s strength lies in its reliance on nationally representative data, which makes its findings applicable to children across all SSA countries. While DHS surveys are conducted in a cross-sectional manner, they may not accurately reflect the true causal relationship between child health and nutrition. Additionally, the data gathered through these surveys is based on self-reported information, which can be influenced by recall bias or social desirability bias, representing a potential limitation of the study.

## Conclusion

The study highlights a concerningly high prevalence of inadequate MDD among children aged 6 to 23 months in SSA, with significant disparities observed between multidimensionally poor and non-poor households. The prevalence of inadequate MDD was highest in Central Africa, while the largest gap between poor and non-poor households was found in East Africa. Key factors contributing to these disparities include women’s education, husbands’ education, the employment status of both parents, household wealth, place of residence (urban vs. rural), family size, and the number of children in the household. These factors were found to either widen or narrow the gap in inadequate MDD between poor and non-poor households.

The findings underscore the urgent need for targeted interventions and policies to address inadequate MDD among young children in SSA. Efforts should focus on reducing poverty, enhancing maternal education, and increasing employment opportunities, particularly for women, by promoting equitable economic prospects. Addressing these underlying factors is crucial to bridging the gap in dietary diversity and improving the nutritional outcomes of children in the region.

## Data Availability

Publicly available datasets were analyzed in this study. This data can be found at: https://dhsprogram.com/.

## References

[1] Eshete T Kumera G Bazezew Y Mihretie A Marie T. Determinants of inadequate minimum dietary diversity among children aged 6–23 months in Ethiopia: secondary data analysis from Ethiopian demographic and health survey 2016. Agric Food Secur. (2018) 7:1–8. doi: 10.1186/s40066-018-0219-8, PMID: 40093350

[2] Organization WH. WHO expert consultation on public health intervention against early childhood caries: report of a meeting, Bangkok, Thailand, 26–28 January 2016. Bangkok, Thailand: World Health Organization (2017).

[3] Organization WH. Infant and young child feeding: Model chapter for textbooks for medical students and allied health professionals. Geneva, Switzerland: World Health Organization (2009). 23905206

[4] Black RE Allen LH Bhutta ZA Caulfield LE De Onis M Ezzati M. Maternal and child undernutrition: global and regional exposures and health consequences. Lancet. (2008) 371:243–60. doi: 10.1016/S0140-6736(07)61690-0, PMID: 18207566

[5] Onyango AW Borghi E de Onis M del Carmen CM Garza C. Complementary feeding and attained linear growth among 6–23-month-old children. Public Health Nutr. (2014) 17:1975–83. doi: 10.1017/S1368980013002401, PMID: 24050753 PMC11108726

[6] Arimond M Ruel MT. Dietary diversity is associated with child nutritional status: evidence from 11 demographic and health surveys. J Nutr. (2004) 134:2579–85. doi: 10.1093/jn/134.10.2579, PMID: 15465751

[7] Mollier L Seyler F Chotte J-L Ringler C. End hunger, achieve food security and improved nutrition and promote sustainable agriculture: SDG 2. Paris, France: ICSU (2017).

[8] Mitchell MK. Nutrition across the life span. Philadelphia, Pennsylvania: WB Saunders (2003).

[9] Organization WH. Nutrition and food safety (NFS) and COVID-19. Geneva, Switzerland: Nutrition and Food Safety (2023).

[10] Kang Y Chimanya K Matji J Garg A Heidkamp R Marshal Q. Determinants of minimum dietary diversity among children aged 6-23 months in 7 countries in east and southern Africa (P10-035-19). Curr Dev Nutr. (2019) 3:nzz034.P10-035-19. doi: 10.1093/cdn/nzz034.P10-035-19, PMID: 39664488

[11] Belete K Daba DB Shallo SA Yebassa MA Danusa KT Gadisa D. Levels of dietary diversity and its associated factors among children aged 6–23 months in West Shoa, Ethiopia: a comparative cross-sectional study. J Nutr Sci. (2022) 11:e20. doi: 10.1017/jns.2022.17, PMID: 35399555 PMC8943564

[12] Dafursa K Gebremedhin S. Dietary diversity among children aged 6–23 months in Aleta Wondo District, southern Ethiopia. J Nutr Metab. (2019) 2019:1–10. doi: 10.1155/2019/2869424, PMID: 31815015 PMC6878804

[13] Demie TG Gesese GT Derseh BT Mruts KB Gebremariam TB. Factors associated with minimum dietary diversity among children aged 6 to 23 months in Debre Berhan town, Central Ethiopia: Community-based Cross-sectional Study. (2021).

[14] Paramashanti BA Huda TM Alam A Dibley M. Trends and determinants of minimum dietary diversity among children aged 6–23 months: a pooled analysis of Indonesia demographic and health surveys from 2007 to 2017. Public Health Nutr. (2021) 25:1956–67. doi: 10.1017/S1368980021004559, PMID: 34743776 PMC9991623

[15] Khanam M Sarker AR. Dietary diversity among children aged 6–23 months in Bangladesh: determinants and inequalities. Bangl Dev Stud. (2023) 44:81–102. doi: 10.57138/HKFC5019

[16] Belay D Aragaw FM Teklu RE Fetene SM Negash W Asmamaw DB. Determinants of inadequate minimum dietary diversity intake among children aged 6–23 months in Sub-Saharan Africa: pooled prevalence and multilevel analysis of demographic and health survey in 33 Sub-Saharan African countries. Front Nutr. (2022) 9:894552. doi: 10.3389/fnut.2022.894552, PMID: 35845763 PMC9284213

[17] Ba D Ssentongo P Gao X Chinchilli V Richie J Maiga M. Prevalence and determinants of meeting minimum dietary diversity among children aged 6–23 months in three Sub-Saharan African countries: the demographic and health surveys, 2019–2020. Front Public Health. (2022) 10:846049. doi: 10.3389/fpubh.2022.846049, PMID: 36081474 PMC9445207

[18] Roy A Hossain MM Hanif AA Khan MS Hasan M Hossaine M. Prevalence of infant and young child feeding practices and differences in estimates of minimum dietary diversity using 2008 and 2021 definitions: evidence from Bangladesh. Curr Dev Nutr. (2022) 6:nzac026. 35415389 10.1093/cdn/nzac026 PMC8992578

[19] Bryce J El Arifeen S Bhutta ZA Black RE Claeson M Gillespie D. Getting it right for children: a review of UNICEF joint health and nutrition strategy for 2006–15. The Lancet. (2006) 368:817–9. 10.1016/S0140-6736(06)69299-4 16950338

[20] Aziz AH Moniruzzaman M. Challenges in Achieving SDG 2-Zero Hunger in Bangladesh: An analytical study on food certainty in Bangladesh from Zakat Perspective. International Journal of Zakat. (2023) 8:43–63.

[21] Gil JD Reidsma P Giller K Todman L Whitmore A Van Ittersum M. Sustainable development goal 2: Improved targets and indicators for agriculture and food security. Ambio. (2019) 48:685–98 30267284 10.1007/s13280-018-1101-4 PMC6509081

[22] Lartey A. End hunger, achieve food security and improved nutrition and promote sustainable agriculture. UN Chronicle. (2015) 51:6–8. doi: 10.18356/5940d90a-en

[23] Arora NK Mishra I. Current scenario and future directions for sustainable development goal 2: A roadmap to zero hunger. Environ Sustain. (2022) 5:129–33. 10.1007/s42398-022-00235-8 PMC9175521 37521584

[24] Wudil AH Usman M Rosak-Szyrocka J Pilař L Boye M. Reversing years for global food security: a review of the food security situation in Sub-Saharan Africa (SSA). Int J Environ Res Public Health. (2022) 19:14836. doi: 10.3390/ijerph192214836 36429555 PMC9690952

[25] Nabiyeva GN Wheeler SM London JK Brazil N. Implementation of sustainable development goal 11 (sustainable cities and communities): initial good practices data. Sustain. (2023) 15:14810.

[26] Kundu S Das P Rahman MA Banna MHA Fatema K Islam MA. Socio-economic inequalities in minimum dietary diversity among Bangladeshi children aged 6–23 months: a decomposition analysis (2022) 12:21712. doi: 10.1038/s41598-022-26305-9, PMC9755277 36522494

[27] Achoki T Miller-Petrie MK Glenn SD Kalra N Lesego A Gathecha GK. Health disparities across the counties of Kenya and implications for policy makers, 1990–2016: a systematic analysis for the global burden of disease study 2016. Lancet Glob Health. (2019) 7:e81–95. doi: 10.1016/S2214-109X(18)30472-8, PMID: 30482677 PMC6293072

[28] Busumtwi-Sam J. Contextualizing human security: a ‘deprivation–vulnerability’approach. Polic Soc. (2008) 27:15–28. doi: 10.1016/j.polsoc.2008.07.002

[29] UNDP. Human development report 2013: the rise of the south: human progress in a diverse world. UNDP: New York, NY (2013).

[30] Powers DA Yoshioka H Yun M-S. Mvdcmp: multivariate decomposition for non-linear response models. Stata J. (2011) 11:556–76. doi: 10.1177/1536867X1201100404

[31] Akombi BJ Agho KE Hall JJ Wali N Renzaho A Merom D. Stunting, wasting and underweight in Sub-Saharan Africa: a systematic review. Int J Environ Res Public Health. (2017) 14:863. doi: 10.3390/ijerph14080863, PMID: 28788108 PMC5580567

[32] Tripathy A Bhati D Srivastava S Mishra PS. Change in socioeconomic inequality in minimum dietary diversity among children aged 6–23 months in India: Evidence from National Family Health Survey. Child Indic Res. (2022) 16:1049–71. doi: 10.1007/s12187-022-10004-y, PMID: 40103751

[33] Sisay BG Afework T Jima BR Gebru NW Zebene A Hassen HY. Dietary diversity and its determinants among children aged 6–23 months in Ethiopia: evidence from the 2016 demographic and health survey. J Nutr Sci. (2022) 11:e88. doi: 10.1017/jns.2022.87, PMID: 36304826 PMC9554528

[34] Gebremedhin S. Core and optional infant and young child feeding indicators in Sub-Saharan Africa: a cross-sectional study. BMJ Open. (2019) 9:e023238. doi: 10.1136/bmjopen-2018-023238, PMID: 30782876 PMC6377519

[35] Sirait ARA Achadi E. Factors associated with minimum dietary diversity among Breas tfed children aged 6-23 months in Indonesia (analysis of Indonesia DHS 2017). Indonesian J Public Health Nutr. (2020) 1:2. doi: 10.7454/ijphn.v1i1.4380

[36] Alemu TG Techane MT Wubneh CA Assimamaw NT Belay GM Tamir TT. Spatial variation and determinates of dietary diversity among children aged 6–23 months in Ethiopia: Spatial and multilevel analysis using Ethiopian demography health survey (EDHS) 2019. Arch Public Health. (2022) 80:152. doi: 10.1186/s13690-022-00905-3 35668474 PMC9169324

[37] Variyam JN Blaylock J Lin BH Ralston K Smallwood D. Mother's nutrition knowledge and children's dietary intakes. Am J Agric Econ. (1999) 81:373–84. doi: 10.2307/1244588

[38] Assefa D Belachew T. Minimum dietary diversity and associated factors among children aged 6–23 months in Enebsie Sar Midir Woreda, East Gojjam, North West Ethiopia. BMC Nutr. (2022) 8:149. doi: 10.1186/s40795-022-00644-2 36539901 PMC9768955

[39] Aemro MMM Birhanu Z Atenafu A. Dietary diversity and meal frequency practices among infant and young children aged 6–23 months in Ethiopia: a secondary analysis of Ethiopian demographic and health survey 2011. J Nutr Metab. (2013) 2013:1–8. doi: 10.1155/2013/782931, PMID: 24455218 PMC3878383

[40] Hailu BA Bogale GG Beyene J. Spatial heterogeneity and factors influencing stunting and severe stunting among under-5 children in Ethiopia: spatial and multilevel analysis. Sci Rep. (2020) 10:1–10. doi: 10.1038/s41598-020-73572-5, PMID: 33009463 PMC7532151

